# Serum microRNA-21 as a Potential Biomarker for Response to Hypomethylating Agents in Myelodysplastic Syndromes

**DOI:** 10.1371/journal.pone.0086933

**Published:** 2014-02-04

**Authors:** Yundeok Kim, June-Won Cheong, Yeo-Kyeoung Kim, Ju-In Eom, Hoi-Kyung Jeung, Soo Jeong Kim, Dohyu Hwang, Jin Seok Kim, Hyeuong Joon Kim, Yoo Hong Min

**Affiliations:** 1 Department of Internal Medicine, Yonsei University College of Medicine, Seoul, Korea; 2 Department of Internal Medicine, Chonnam National University College of Medicine, Jeollanam-do, Korea; 3 Medical Research Center, Yonsei University College of Medicine, Seoul, Korea; Cincinnati Children’s Hospital Medical Center, United States of America

## Abstract

Identification of biomarkers that predict responses to hypomethylating agents (HMAs) will allow optimal strategies for epigenetic therapy in myelodysplastic syndromes (MDS) to be established. Serum miR-21 was quantitatively measured in 58 MDS patients treated with HMAs and 14 healthy controls. Serum miR-192 was an internal control, and diagnostic performance was evaluated according to receiver operating characteristics (ROCs). ROC analysis indicated that serum miR-21 levels differentiated responders from non-responders with an area under the curve of 0.648 (95% confidence, 0.49 to 0.72). The baseline level of serum miR-21 was significantly lower in the responder group than in the non-responder group (*P* = 0.041). The overall response rate (ORR) of the high miR-21 group was significantly lower than that of the low miR-21 group (41.2 *vs.* 73.2%, *P = *0.021). Progression-free survival (PFS) was significantly inferior in the high group versus the low group (14.0 *vs.* 44.5 months, *P* = 0.001). Multivariate analyses revealed that the initial serum miR-21 level (*P* = 0.001) and circulating blasts (*P* = 0.007) were prognostic factors for PFS. Serum miR-21 level was significantly associated with ORR and PFS in MDS patients treated with HMAs. Although validation with a large prospective study is required, serum miR-21 is a potential biomarker of epigenetic therapy in MDS patients.

## Introduction

Myelodysplastic syndromes (MDS) are a heterogeneous group of clonal disorders characterized by bone marrow failure, dysplasia of one or more of the myeloid blood cell lineages, and an increased risk of developing acute myeloid leukemia [1]. Recently, epigenetic therapy with hypomethylating agents (HMAs) has demonstrated clinical effectiveness for MDS [2]–[4]. For instance, azacitidine (AZA) can improve hematologic parameters, delay transformation into acute myeloid leukemia (AML), and improve quality of life and survival in comparison with supportive care [3]. However, response to HMA differs between patients, with overall response rates ranging from 40 to 60% [5], [6]. If response to HMA can be predicted, treatment outcomes can be improved by selecting MDS patients in whom HMA are likely to be beneficial and by developing risk-adapted therapeutic strategies. Unfortunately, biomarkers that predict response or survival following treatment with AZA or decitabine (DAC) remain largely unidentified and debated in patients with MDS. Clinical parameters, including bone marrow blasts, abnormal karyotype, World Health Organization (WHO) classification-based prognostic scoring system (WPSS), and interval from diagnosis to the initiation of HMA, were reported to be associated with response to HMA [7], [8]. However, these findings were not confirmed in other studies [9]. Further, associations with survival outcomes were not demonstrated. Molecular parameters, such as mutations in *TET2* [10], *DNMT3A* [11], or *ASXL1* [11], induction of p53-inducible ribonucleotide reductase [12], reactivation of *phosphoinositide phospholipase Cβ* gene [13], and gene methylation profile [14], correlated with clinical response to HMA.

MicroRNAs (miRs) are short noncoding RNAs that modulate gene expression by negatively regulating stability or translational efficiency of the target mRNA [15]–[17]. MiRs have critical roles in developmental processes, hematopoiesis, cell differentiation, proliferation, and apoptosis [18]–[20]. In addition, deregulation of miRs has been shown to contribute to the development of a variety of tumors, such as leukemia [21], [22], neuroblastoma [23], breast cancer [18], and lung cancer [24]. Aberrant expression of miRs was shown to have crucial roles in tumor progression and development of chemoresistance [25]–[28]. For these reasons, miRNA expression analyses have been applied to tumor diagnosis, treatment, and prognostic prediction [27]–[29].

MiRs can be consistently detected and quantitatively measured by real-time polymerase chain reaction in plasma and serum samples, which are easily obtained from cancer patients [29]–[32]. Thus, circulating miRs are potentially suitable biomarkers for a variety of cancers. In this study, we evaluated the potential of serum miR-21 as a biomarker for predicting response to HMA in MDS patients. MiR-21, a representative oncogenic miRNA [29], [33]–[35], has prognostic significance in many cancers [27], [28] and can modulate sensitivity to chemotherapeutic agents [24]–[26], [28]. There is increasing evidence that circulating miR-21 may be a useful biomarker in various cancers [29], [30], [36]. For example, high levels of serum miR-21 in patients with diffuse large cell lymphoma were related to lymphoma recurrence and patient survival [36]. Further, plasma miR-21 expression was related to the sensitivity of non-small cell lung cancer to platinum-based chemotherapy [24].

In the present study, we demonstrate for the first time that serum miR-21 level is a potential biomarker associated with clinical response to epigenetic treatment in MDS. Progression-free survival (PFS) following HMA treatment was significantly shorter in patients with high serum miR-21 levels. Circulating miR-21 should be further validated as a biomarker in MDS through a prospective study with a large cohort.

## Methods

### Patients and Samples

We analyzed clinical records and samples from 58 patients with MDS treated with HMA between January 2006 and December 2011 at Yonsei University Severance Hospital and Chonnam National University Hospital. Forty-six patients received AZA, and 12 received DAC. For each cycle, AZA and DAC were administered subcutaneously at a dose of 75 mg/m^2^/day for 7 days and intravenously at a dose of 20 mg/m^2^/day for 5 days, respectively. HMAs were administered until disease progression or intolerable toxicities developed. All patients were enrolled onto protocols approved by the Institutional Review Board of Severance Hospital and provided written informed consent in accordance with the Declaration of Helsinki.

### Responses

Response was assessed by modified International Working Group (IWG2006) response criteria [37]. Overall response rate (ORR) included rates for complete response (CR), marrow CR (mCR), partial response (PR), and hematologic improvement (HI). Overall survival (OS) and PFS were measured from the date of initiation of HMA to death or censored at the time of last contact and to progression of disease or leukemic transformation, respectively.

### MicroRNA Quantification by Quantitative Real-time Polymerase Chain Reaction (qRT-PCR)

The purity and concentration of RNA isolated using mirVana PARIS kit (Ambion Inc., Austin, TX) were evaluated with a NanoDrop ND-1000 spectrophotometer (NanoDrop Technologies, Rockland, DE, USA). Serum miR-21 quantification was performed with the SYBR-Green qRT-PCR assay on an ABI 7500 RT-PCR instrument (Applied Biosystems, Foster City, CA). The amplification profile was as follows; denaturing at 95°C for 10 min, 40 cycles at 95°C for 15 sec, 60°C for 30 sec, and 72°C for 1 sec. All reactions were run in triplicate. Relative levels of miRs were quantified by the comparative CT (2^−ΔΔCT^) method, in which ΔCt = mean Ct_miRNA_−mean Ct_internal control_. The cycle threshold (Ct) was defined as the number of cycles required for the fluorescent signal to cross the threshold in qRT-PCR.

### Evaluation of Internal Controls for Serum miR-21 Quantification

To select a reliable internal reference miR, we evaluated the abundance and stability of three endogenous control miRs, miR-192, miR-16, and miR-93, in the sera of 16 MDS patients and 8 healthy donors.^ 30,32,38^ Gene expression levels were analyzed in triplicate and comparative CT method algorithms. If expression levels were equivalent between groups, the stabilities of candidate reference genes were compared by the *geNorm* software (version 3.4) method [39].

### Statistical Analysis

Characteristics of patients are presented as median and range for continuous factors and frequencies for discrete factors. The χ^2^-test was used for comparison of categorical variables such as prognostic factors for treatment response. The sensitivity and specificity of the optimum cut-off point were defined as those values that maximized the area under the receiver operating characteristic curve (AUC). The optimal cut-off point for miR-21 was determined by the Youden index (sensitivity+specificity-1). The OS and PFS results in low and high miR-21 group were estimated by the Kaplan-Meier method and compared by the log-rank test. Multivariate analyses were performed by Cox proportional-hazards regression analysis. *P* value less than 0.05 denoted statistical significance. All analyses were performed with the SPSS 18.0 statistical package (SPSS Inc., Chicago, IL).

## Results

### Patient Characteristics

The study population included 58 patients (38 males and 20 females) with a median age of 67 years (range, 35–83 years). The characteristics of the patients are summarized in Table 1. Diagnosis according to WHO criteria included five (8.6%) cases of refractory anemia (RA) or RA with ringed sideroblasts, 16 (27.6%) cases of RA with multilineage dysplasia, 11 (19.0%) cases of RA with excess blasts-1 (RAEB-1), 20 (34.5%) cases of RAEB-2, and six (10.3%) of MDS-unclassified. Median time from the diagnosis to HMA initiation was 0.7 months (range, 0–5.8 months). Cytogenetic risk was good in 34 (58.6%), intermediate in eight (13.8%), and poor in 8 (13.8%) patients. Karyotyping was unsuccessful in eight cases. The median number of cycles administered was six (range, 1–16). IPSS was low in two (3.4%), intermediate-1 (Int-1) in 23 (39.7%), intermediate-2 (Int-2) in 19 (32.8%), high in six (10.3%), and undetermined in eight (13.8%) cases. Thirty-three (56.9%) cases were transfusion-dependent. WPSS risk was low in four (6.9%), intermediate in 15 (25.9%), high in 26 (44.8%), and very high in five (8.6%) patients. Forty-six (79.3%) patients received AZA and 12 (20.7%) received DAC. None of the patients received chemotherapy or hematopoietic stem cell transplantation prior to the study.

**Table 1:** Overall characteristics of patients and stratification according to serum miR-21 level.

			Serum miR-21		
Variable		Overall (n = 58)	Low (n = 41)	High (n = 17)	P-value
Age (y), median(range)		67 (35–83)	67 (36–83)	65 (35–78)	0.950
Male, n(%)		38 (65.5)	29 (70.7)	9 (52.9)	0.194
WHO subtype, n(%)	RA/RARS	4 (6.9)/1(1.7)	3 (7.3)/0	1 (5.9)/1(5.9)	0.109
	RCMD	16 (27.6)	10 (24.4)	6 (35.3)	
	RAEB-1	11 (19.0)	5 (12.2)	6 (35.3)	
	RAEB-2	20 (34.5)	18 (43.9)	2 (11.8)	
	MDS-U	6 (10.3)	5 (12.2)	1 (5.9)	
Karyotype risk, n(%)^a^	Good	34 (58.6)	26 (63.4)	8 (47.1)	0.207
	INT	8 (13.8)	6 (14.6)	2 (11.8)	
	Poor	8 (13.8)	4 (9.8)	4 (23.5)	
	N/A	8 (13.8)	5 (12.2)	3 (17.6)	
Hemoglobin (g/dL), median(range)		8.4 (4.8–12.4)	8.5(4.8–12.4)	8.0 (5.5–10.5)	0.225
Neutrophil (x10^9^/L), median(range)		0.93 (0.05–41.15)	0.91 (0.05–41.15)	1.04 (0.16–4.76)	0.701
Platelets (x10^9^/L), median(range)		72 (5–543)	75 (6–548)	69 (5–177)	0.898
PB blast (%), median(range)		0, (0–12)	0, (0–5)	0 (0–12)	0.424
BM blast (%), median(range)		5.1 (0–18.8)	2.0 (0–18.8)	0.2 (0–18.4)	0.173
Interval from diagnosis to HMA therapy, months, median(range)		0.7 (0–5.8)	0.9 (0–5.8)	0.4 (0–5.2)	0.457
IPSS, n(%)	Low	2 (3.4)	1 (2.4)	1 (5.9)	0.715
	INT-1	23 (39.7)	18 (43.9)	5 (29.4)	
	INT-2	19 (32.8)	13 (31.7)	6 (35.3)	
	High	6 (10.3)	4 (9.8)	2 (11.8)	
	N/A	8 (13.8)	5 (12.2)	3 (17.6)	
WPSS,n(%)	Low	4 (6.9)	3 (7.3)	1 (5.9)	0.870
	INT	15 (25.9)	12 (29.3)	3 (17.6)	
	High	26 (44.8)	18 (43.9)	8 (47.1)	
	Very high	5 (8.6)	3 (7.3)	2 (11.8)	
	N/A	8 (13.8)	5 (12.2)	3 (17.6)	
Transfusion dependence, n(%)^b^		33 (56.9)	22 (53.7)	11 (64.7)	0.439
HMA treatment, n(%)	Azacitidine	46 (79.3)	33 (80.5)	13 (76.5)	0.733
	Decitabine	12 (20.7)	8 (19.5)	4 (23.5)	
Serum miR-21 value, median(range)		1.0010(0.2287–2.1946)	0.8290(0.2287–1.2613)	1.7389(1.4319–2.1946)	<0.001
AlloHCT after HMA therapy, n(%)		11 (19.0)	9 (22.0)	2 (11.8)	0.372
Follow-up months,median(range)		15.4 (0.7–66.4)	14.9 (3.7–66.4)	17.1 (0.7–46.0)	0.871
Alive, n(%)		34 (58.6)	25 (61.0)	9 (52.9)	0.484
Leukemic transformation, n(%)		1 (1.7)	1 (2.4)	0	0.184

Abbreviations: miR-21, microRNA-21; WHO, World Health Organization; RA, refractory anemia;

RARS, RA with ring sideroblasts; RCMD, refractory cytopenia with multilineage dysplasia; RCMD-RS, RCMD with ring sideroblasts; RAEB, refractory anemia with excess blasts; MDS-U, myelodysplastic syndromes unclassifiable; PB, peripheral blood; BM, bone marrow; HMA, hypomethylating agent; IPSS, International Prognostic Scoring System; NA, not available; WPSS, WHO classification-based Prognostic Scoring System; alloHCT, allogeneic hematopoietic stem cell transplant, INT, intermediate.

a Cytogenetic: Good = normal, -Y alone, del(5q) alone, del(20q) alone; Poor = complex (≥3 abnormalities) or chromosome 7 anomalies; Intermediate = other abnormalities.

b At least 2 units of red blood cell transfusion during 8 weeks prior to HMA initiation.

### Selection of the Internal Control for Serum miR-21 Quantification

**Figure 1:**
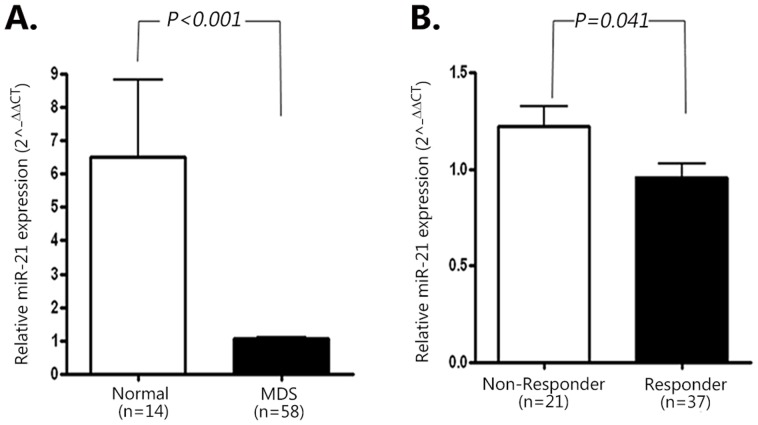
Expression and stability of reference gene candidates in the sera of healthy donors and patients with MDS. (A) Quantification (Ct) of candidate reference miRNAs (miR-192, miR-16, and miR-93) in serum samples of healthy donors and MDS patients corrected for efficiency and two interpolate controls are shown. *Box plots* represent lower and upper quartiles with the median depicted with a horizontal line. Whiskers depict the 10th and 90th percentiles. Differences in serum levels of candidate miRNAs were not found between healthy donors (white box), MDS patients prior to HMA therapy (gray box), and MDS patients treated with four courses of HMA therapy (black box). (B) Average expression stability values for candidate reference miRs in MDS patients, which were calculated by the *geNorm* algorithm, are shown as a bar graph and with actual values. High expression stability is indicated by a low stability value. MDS, myelodysplastic syndromes; miRNAs, microRNAs; HMA, hypomethylating agents.

In order to select a reliable internal control for quantification of serum miR-21 in MDS, we examined miR-192, miR-16, and miR-93 levels by qRT-PCR as described in Design and Methods. Expression levels of serum miR-16, miR-93, and miR-192 did not differ between normal donors, MDS patients prior to HMA therapy, and MDS patients who received four cycles of HMA (Figure 1A). However, the median Ct values of miR-16 (33.1; range, 32.0–34.1) and miR-93 (31.7; range, 30.5–33.0) were high in normal donors, MDS patients prior to HMA therapy, and MDS patients who received four cycles of HMA. In contrast, the Ct values of serum miR-192 were low in all three groups (median 25.9; range 25.6–26.4). Stability values of the three candidate reference miRNAs were calculated by *geNorm* analyses. *GeNorm* analyses showed that *M* values of miR-16 and miR-93 were 0.007 and 0.009, respectively. miR-192 had the lowest *M* value of 0.003, indicating that miR-192 was stably expressed in serum (Figure 1B). Thus, miR-192 was selected as an internal control miRNA for quantifying serum miR-21 in MDS patients in this study.

### Clinical Characteristics according to Serum miR-21 Levels

**Figure 2:**
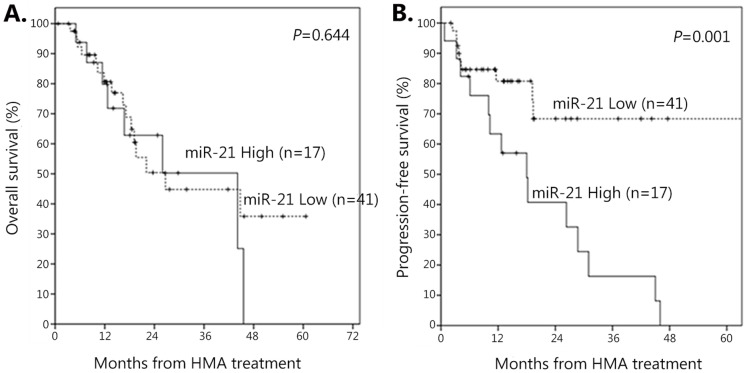
Differences in baseline serum miR-21 expression levels. (A) Difference in serum miR-21 levels between healthy donors and patients with MDS prior to HMA therapy. Expression levels of serum miR-21 were normalized to the reference gene, miR-192, which was selected as described in Design and Methods. (B) Difference in baseline serum miR-21 levels between responders to HMA therapy and non-responders. Bar graph and whisker indicate the median value and standard deviation of serum miR-21 levels, respectively. Significance is indicated by the *P* value.

Serum miR-21 expression levels were examined by qRT-PCR in 58 patients with MDS and 14 healthy volunteers. The relative level of serum miR-21 expression was significantly higher in healthy donors than in MDS patients at diagnosis (4.2047±4.1479 vs. 0.9414±0.8690, *P*<0.001, Figure 2A). Because serum miR-21 levels showed heterogeneity among patients, we arbitrarily classified the patients with serum miR-21 levels (2^−ΔΔCT^) greater than the cut-off value of 1.2613 as the high-miR-21 group (n = 17) and the remaining patients as the low-miR-21 group (n = 41) as described in Design and Methods. The median value of serum miR-21 in the high-miR-21 group was significantly higher than that of the low-miR-21 group (1.7380 *vs.* 0.8290, *P*<0.001). There were no differences in patient age, sex, baseline hemoglobin levels, neutrophils, platelet counts, percentages of blasts in the peripheral blood or bone marrow, interval from diagnosis to HMA initiation, and transfusion dependency (≥4 RBC units/8 weeks) between the high-miR-21 and low-miR-21 groups (Table 1). No significant differences in WHO diagnosis, cytogenetic risk, IPSS risk categories, and WPSS risk were found between the high-miR-21 and low-miR-21 groups. The relative proportions of patients who received AZA or DAC as HMA treatment were comparable between the two groups (Table 1). Follow-up period and the proportion of patients who received allogeneic hematopoietic stem cell transplantation after HMA therapy were similar between the groups.

### Relationship between Serum miR-21 Levels and Response to HMA

**Figure 3:**
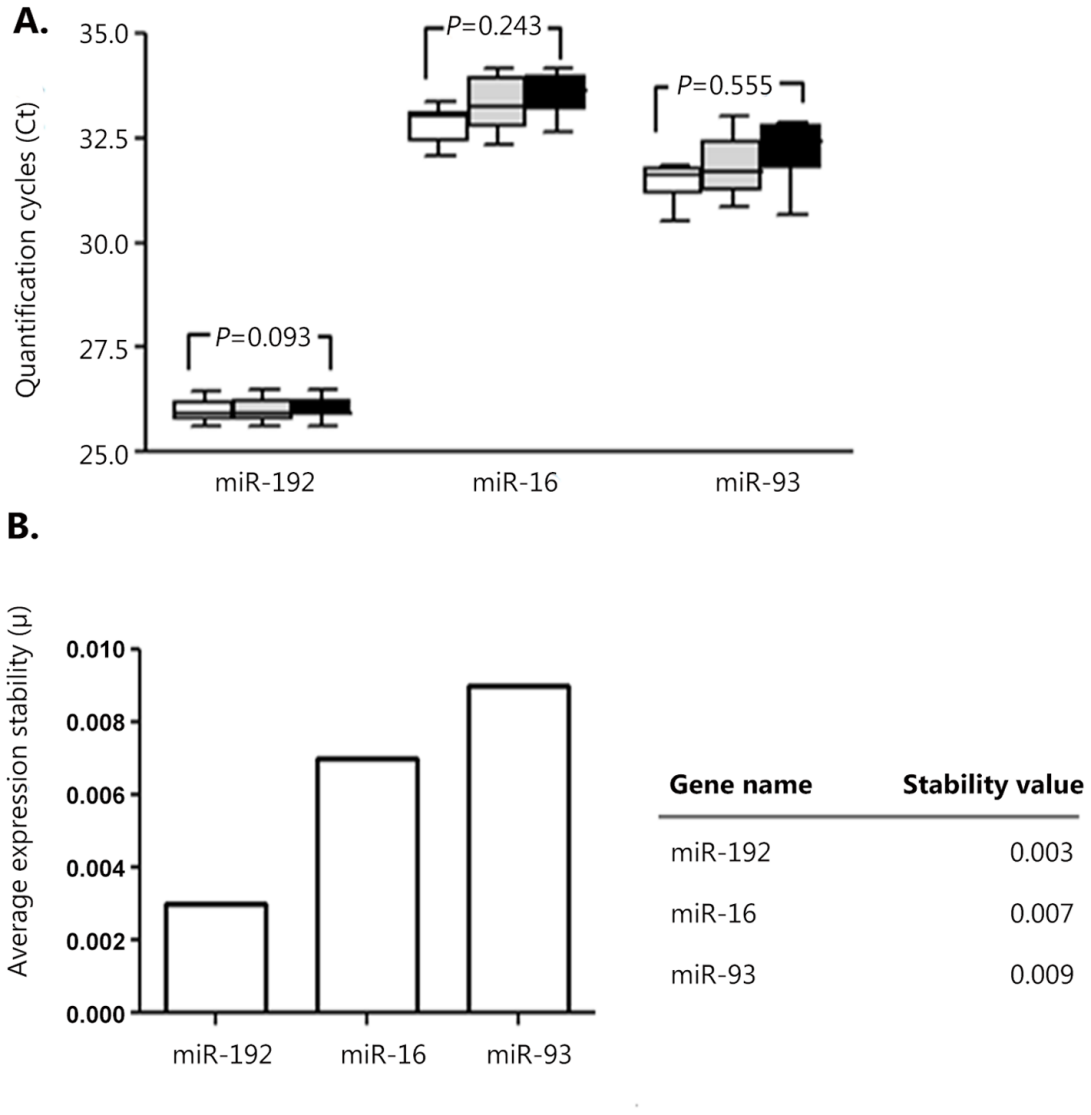
Receiver operating characteristics (ROCs) curve analysis for the diagnostic value of miR-21. The area under the ROC curve (AUC) was 0.648 (95% CI: 0.49 to 0.72).

Response to HMA is summarized in Table 2. The ORR, which included CR, mCR, PR, and SD with HI, was 63.8%. CR was achieved in two cases (3.4%), PR in two (3.4%), mCR in three (5.2%), and SD with HI in 30 cases (51.7%). The median time to response was 4.5 months (range, 0.2–50.5 months), and the median duration of response was 39.1 months (range, 28.5–49.8 months). ROC curve analyses were performed to evaluate whether serum miR-21 is a potential marker for predicting response to HMA. ROC analysis indicated that serum miR-21 differentiated responders from non-responders with an AUC of 0.648 (95% confidence interval [CI], 0.49 to 0.72; Figure 3). At the cut-off value of 1.2613 for serum miR-21 level (2^−ΔΔCT^), the optimal sensitivity and specificity were 83.3% (95% CI: 68.6–93.3%) and 45.8% (95% CI: 25.5–67.1%), respectively.

**Table 2:** Response to HMA therapy according to serum miR-21 level.

			Serum miR-21		
		Overall (n = 58)	Low (n = 41)	High (n = 17)	P-value
HMA cycles administrated (range)		6 (1–16)	7 (1–12)	6 (3–16)	0.474
HMA cycles for best response (range)		4 (2–14)	4 (2–12)	4 (2–14)	0.632
Time to response, months (range)		4.5 (1.0–50.5)	4.3 (1.0–50.5)	5.1 (1.0–23.8)	0.956
Response by IWG 2006 criteria, n (%)	CR/PR	4 (6.8)	3 (7.3)	1 (5.9)	0.542
HMA cycles administrated (range)	mCR	3 (5.2)	3 (7.3)	0	
	SD with HI	30 (51.7)	24 (58.5)	6 (35.3)	
	SD without HI	14 (24.1)	6 (14.6)	8 (47.1)	
	PD/Failure	7 (12.0)	5 (12.1)	2 (11.8)	
	CR/PR	4 (6.8)	3 (7.3)	1 (5.9)	
	mCR	3 (5.2)	3 (7.3)	0	
ORR (CR+PR+mCR+SD with HI)		37 (63.8)	30 (73.2)	7 (41.2)	0.012
Response duration, months (range)		39.1 (28.5–49.8)	47.2 (36.6–58.0)	18.1 (7.6–28.6)	0.067

Abbreviations: miR-21, microRNA-21; HMA, hypomethylating agent; IWG, International Working Group; CR, complete response; PR, partial response; mCR, marrow CR; SD, stable disease; HI, hematologic improvement; PD, progressive disease; ORR, overall response rate.

Results are shown as n (%) or median value.

Response to HMA according to serum miR-21 level is shown in Figure 2B and Table 2. The baseline level of serum miR-21 was significantly lower in the responder group than in the non-responder group (0.9417±0.6349 vs. 1.1443±0.7707, *P* = 0.041, Figure 2B). ORR in the high-miR-21 group was significantly lower than in the low-miR-21 group (41.2% *vs.* 73.2%, *P = *0.012). However, the number of HMA cycles administered was comparable between the two groups. The number of HMA cycles required for achieving best response was also similar between the groups. The frequency of MDS patients who did not respond, which included cases of SD without HI, progression of disease, or AML transformation, was higher in the high-miR-21 group (10/17, 58.9%) compared to the low-miR-21 group (11/41, 26.7%, *P* = 0.012). The median duration of response in the high-miR-21 group was shorter (18.1 months, range, 7.6–28.6 months) than in the low-miR-21 group (47.2 months, range, 36.5–58.0 months), although statistical significance was not observed (*P* = 0.067, Table 2). In addition to serum miR-21 levels, cytogenetic risk (*P* = 0.029) and the presence of circulating blasts at diagnosis (*P* = 0.040) were significantly associated with low response rate to HMA (Table 3). However, neither patient age, baseline neutrophils, platelet counts, number of cases with cytopenia, percentage of bone marrow blasts, IPSS risk, WPSS risk, nor red blood cell transfusion dependence were associated with response to HMA. Multivariate Cox regression indicated that only cytogenetic risk was significantly correlated with ORR (HR = 0.575; 95% CI, 0.340 to 0.974; *P* = 0.040) (Table 4).

**Table 3:** Univariate analysis for overall response rate, overall survival, and progression-free survival.

		ORR, n (%)	*P* value	OS, median (mo)	*P* value	PFS, median (mo)	*P* value
Age, years	<65	17 (63.0)	0.772	22.1	0.942	24.7	0.727
	≥65	20 (66.7)		26.7		44.1	
Neutrophils (×10^9^/L)	<1.0	21 (70.0)	0.263	32.6	0.596	44.1	0.118
	≥1.0	15 (55.6)		30.6		18.4	
Hemoglobin (g/dL)	<9	22 (64.7)	0.863	19.1	0.058	45.0	0.980
	≥9	15 (62.5)		44.7		28.7	
WHO subtype	RCMD/RCMD-RS	11 (61.1)	0.334	44.1	0.278	31.0	0.329
	RAEB-1	6 (54.5)		12.6		28.7	
	RAEB-2	11 (61.1)		22.0		12.7	
	RA/RARS/MDS-U	9 (81.8)		26.6		38.6	
Cytopenia	0–1 lineage	11 (64.7)	0.876	39.7	0.257	24.7	0.929
	2–3 lineage	25 (62.5)		28.6		29.7	
Marrow blasts (%)	<15	33 (63.5)	0.878	16.0	0.129	12.3	0.947
	≥15	4 (66.7)		5.0		5.3	
Presence of circulating blasts	Yes	2 (28.6)	0.040	16.7	0.200	8.2	<0.001
	No	35 (68.6)		26.7		29.7	
IPSS Cytogenetic	Good	27 (79.4)	0.029	26.0	0.110	13.6	0.429
	Intermediate	3 (37.5)		22.1		9.2	
	Poor	4 (50.0)		12.7		8.2	
	N/A	3 (37.5)		45.6		16.1	
IPSS	Low/Int-1	17 (68.0)	1.000	26.6	0.125	31.0	0.995
	Int-2/High	17 (68.0)		19.1		28.7	
WPSS	Low/Int	14 (73.6)	0.500	44.1	0.057	31.0	0.508
	High/Very high	20 (64.5)		19.1		28.7	
RBC transfusion dependency	Yes	21 (63.6)	0.977	44.1	0.408	24.7	0.989
	No	16 (64.0)		22.1		45.5	
AlloHCT after HMA therapy	Yes	31 (83.7)	0.478	33.6	0.537	35.3	0.330
	No	6 (16.3)		31.7		29.3	
Serum miR-21 level	Low	30 (73.2)	0.021	26.7	0.644	45.5	0.001
	High	7 (41.2)		44.1		14.0	

Abbreviations: ORR, overall response rate; OS, overall survival; PFS, progression-free survival; RA, refractory anemia; RARS, RA with ring sideroblasts; RCMD, refractory cytopenia with multilineage dysplasia; RCMD-RS, RCMD with ring sideroblasts; RAEB, refractory anemia with excess blasts; MDS-U, myelodysplastic syndromes unclassifiable; IPSS, International Prognostic Scoring System; NA, not available; WPSS, WHO classification-based Prognostic Scoring System; RBC, red blood cell; alloHCT, allogeneic hematopoietic stem cell transplant; miR-21, microRNA-21.

**Table 4:** Multivariate analysis for overall response to HMA and progression-free survival.

Variable	ORR			PFS		
	HR	95% CI	*P* value	HR	95% CI	*P* value
IPSS cytogenetic	0.575	0.340–0.974	0.040			
Circulating blasts	0.238	0.038–1.509	0.128	5.028	1.569–16.118	0.007
Neutrophil counts				1.957	0.790–4.847	0.147
Serum miR-21 level	0.302	0.084–1.086	0.067	4.189	1.757–9.989	0.001

Abbreviations: ORR, overall response rate; PFS, progression-free survival; HR, hazard ratio; CI, confidence interval; IPSS, International Prognostic Scoring System; miR-21, microRNA-21.

### Survival According to Serum miR-21 Levels

**Figure 4:**
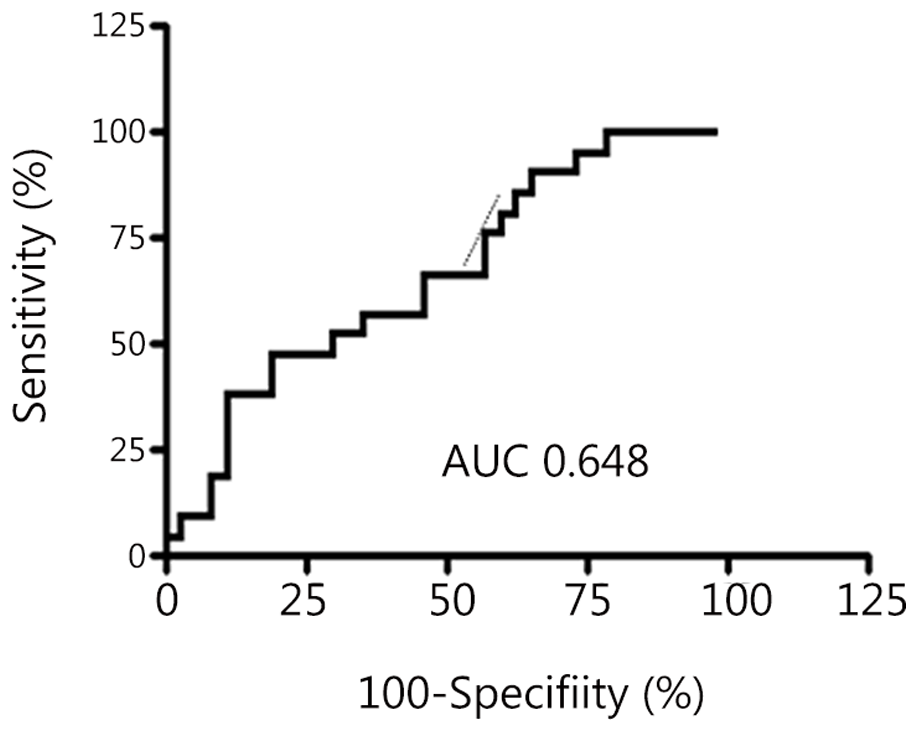
Kaplan-Meier curves for overall survival and progression-free survival comparisons. (A) Overall survival and (B) progression-free survival according to baseline serum miR-21 levels in patients with myelodysplastic syndromes treated with hypomethylating agents.

The median follow-up period was 15.4 months (range, 0.7–66.4 months), and 34 patients survived. Figure 2 shows Kaplan-Meier curves for OS and PFS stratified according to baseline serum miR-21 level. The median OS for the high-miR-21 group (22.6 months, 95% CI 12.4–32.7 months) was shorter than that of the low-miR-21 group (45.0 months, 95% CI 15.3–74.6 months), although it was not statistically different (*P* = 0.644, Figure 4A). None of the clinical parameters that we analyzed correlated with OS in HMA-treated MDS patients (Table 3). However, median PFS was significantly shorter in patients with high serum miR-21 than in patients with low serum miR-21 (14.0 *vs.* 44.5 months, *P* = 0.001, Table 3 and Figure 4B). The one-year PFS rate was significantly higher in the low-miR-21 group compared with the high-miR-21 group (80.8% *vs.* 57.0%, *P* = 0.001). Eleven patients received allogeneic hematopoietic stem cell transplantation after HMA treatment. However, censoring at the time of transplant did not affect PFS outcomes (*P* = 0.330, data not shown). In addition to serum miR-21 level, the presence of circulating blasts at diagnosis was associated with inferior PFS (Table 3). Multivariate analyses revealed that circulating miR-21 (HR = 4.189; 95% CI, 1.757 to 9.989; *P* = 0.001) and the presence of circulating blasts at diagnosis (HR = 5.028; 95% CI, 1.569 to 16.118; *P* = 0.007) remained prognostic factors for PFS (Table 4).

## Discussion

Although HMA have considerably improved treatment outcomes for patients with MDS [2], responses and clinical outcomes differ between patients [2], [4], [10], [40], [41]. Therefore, identification of biomarkers that predict response to epigenetic therapy will undoubtedly improve results of HMA treatment in MDS, making individualized risk-adapted therapy possible. In this study, we demonstrated for the first time that serum miR-21 was significantly associated with response rate to HMA and PFS in patients with MDS.

Currently, there are no clinical parameters or biomarkers that can consistently predict response to HMA therapy or survival benefit in patients with MDS. Several variables have been reported to be associated with response to HMA [7], [8], although some of these findings were not confirmed in other series [9]. Previous low-dose cytarabine therapy [7], [8], BM blasts [7], [8], abnormal karyotype [8], WPSS risk [41], doubling of the platelet count after the first cycle of AZA [42], and the time interval from diagnosis to initiation of HMA therapy [8] were associated with overall response rate to AZA. Chromosome 7 abnormalities were associated with a higher likelihood of response to DAC [4], [43]. The putative mechanism of action of HMA is inhibition of DNA methyltransferase 1, leading to CpG dinucleotide hypomethylation [44]. However, gene methylation patterns before or during treatment did not correlate with clinical response to HMA [15], [45]. Recently it was demonstrated that mutations in the *ten-eleven-translocation 2* (*TET2*) [10], [12], *DNMT3A* [11], [12], and *ASXL1* genes [12] predicted response to AZA, although these mutations did not impact response duration or overall survival [46].

Because of their high stability in blood [30], [31], circulating miRs could be potentially utilized as noninvasive biomarkers for diagnosis or prognosis in a variety of diseases. Furthermore, serum or plasma miRs are more desirable than tissue miRs as biomarkers, because they can be harvested easily and regularly monitored [29]–[31]. Peripheral cytopenia is usually present in MDS, and it is uncertain which cells are suitable targets for MDS analysis. For those reasons, serum or plasma can be expected to be better sources for miR assays in patients with MDS. However, few studies have evaluated the prognostic relevance of miRs in MDS patients. Recently, it was shown that circulating let-7a and miR-16 were downregulated in MDS. Levels of let-7a and miR-16 were significantly associated with PFS and OS [38], supporting the view that circulating miRs are potential biomarkers in MDS. Quantitative measurement of circulating miRs as a reliable biomarker assay requires normalization to enable comparisons of miRNA levels across different samples [41]. In this study, miR-192 was identified through *geNorm* analysis as a suitable internal reference gene for qPCR analysis of serum miRs in MDS patients and healthy controls. In current study, we found low serum levels of miR-21 in MDS patients compared with healthy individuals. Further studies are necessary to evaluation whether the reduced serum miR-21 level returns to normal levels after HMT.

Because of increasing evidence that circulating miR-21 may be a useful biomarker for various cancers [29], [30], [36], we examined if miR-21 is abnormally expressed in the sera of patients with MDS and associated with response to HMA. Our study revealed that serum miR-21 levels were significantly lower in MDS patients compared to healthy controls. Previously Pons *et al* observed higher levels of miR-21 at the cellular level in bone marrow and peripheral blood samples from MDS patients compared with normal donors [47]. Although we could not evaluate miR-21 simultaneously in sera, peripheral blood, and bone marrow cells, these contradictory findings may be due to differences in the type of sample examined in the miR-21 assay.

ROC analyses performed to assess the diagnostic power of serum miR-21 yielded an AUC of 0.648 with 83.3% sensitivity and 45.8% specificity for discriminating responders to HMA from non-responders. Our study revealed that the response rate to HMA was significantly lower in patients with high levels of serum miR-21 compared with patients with low levels of serum miR-21. In addition to serum miR-21, cytogenetic risk and the presence of circulating blasts at diagnosis were observed to be highly associated with poor response to HMA in our study. The time interval from diagnosis to the initiation of HMA therapy did not affect the response rate to HMA. Importantly, PFS was significantly longer in HMA-treated patients with low serum miR-21 compared to HMA-treated patients with high serum miR-21. In multivariate analysis, serum miR-21 level remained an independent predictor of PFS in patients treated with HMA.

The basis for the poor response of patients with high serum miR-21 to HMA remains to be elucidated. Abnormal expression of miR-21 may be related to transcriptional and post-transcriptional regulation [48]. miR-21 regulates cell proliferation, differentiation, and apoptosis by regulating target genes, including tropomyosin 1 (*TPM1*), programmed cell death gene 4 (*PDCD4*), transforming growth factor (TGF-β) pathway genes, *Bcl-2*, and phosphatase and tensin homolog (*PTEN*) [33]–[35], [49]. Activated AKT has been linked to AZA responsiveness [13]. Based on these findings, miR-21 may impact the sensitivity of “MDS cells” to AZA by regulating PTEN/PI3K/AKT signaling. However, the mechanisms regulating miR-21 expression in sera and the functional roles of circulating miR-21 in MDS, especially in relation to drug sensitivity, remain unclear. Based on these findings, further exploration of molecular mechanism is needed.

Although we examined a retrospectively small number of patients, serum miR-21 level could be serve as a potential biomarker for predicting response to epigenetic treatment in patients with MDS. PFS was significantly associated with serum miR-21 levels in MDS patients treated with HMA. The clinical potential of serum miR-21 as a biomarker in MDS should be validated in a prospective study that includes a large cohort of patients. Our findings may help clinicians identify patients who are most likely to benefit from epigenetic therapy and may facilitate development of risk-adaptive therapeutic strategies for MDS.
